# Development of a screening tool to identify female survivors of gender-based violence in a humanitarian setting: qualitative evidence from research among refugees in Ethiopia

**DOI:** 10.1186/1752-1505-7-13

**Published:** 2013-06-12

**Authors:** Andrea L Wirtz, Nancy Glass, Kiemanh Pham, Amsale Aberra, Leonard S Rubenstein, Sonal Singh, Alexander Vu

**Affiliations:** 1 Department of Epidemiology, Center for Public Health and Human Rights, Johns Hopkins Bloomberg School of Public Health, Baltimore, USA; 2 Department of Emergency Medicine, Johns Hopkins Medical Institutes, Baltimore, USA; 3 Johns Hopkins School of Nursing, Baltimore, USA; 4 Department of International Health, Johns Hopkins School of Public Health, Baltimore, USA; 5 University of Washington School of Law, Seattle, USA; 6 Department of General Internal Medicine, Johns Hopkins Medical Institutes, Baltimore, USA

**Keywords:** Refugee, Displacement, Conflict, Gender-based violence, Sexual violence, Reproductive health, Ethiopia

## Abstract

**Background:**

High levels of gender-based violence (GBV) persist among conflict-affected populations and within humanitarian settings and are paralleled by under-reporting and low service utilization. Novel and evidence-based approaches are necessary to change the current state of GBV amongst these populations. We present the findings of qualitative research, which were used to inform the development of a screening tool as one potential strategy to identify and respond to GBV for females in humanitarian settings.

**Methods:**

Qualitative research methods were conducted from January-February 2011 to explore the range of experiences of GBV and barriers to reporting GBV among female refugees. Individual interview participants (n=37) included female refugees (≥15 years), who were survivors of GBV, living in urban or one of three camps settings in Ethiopia, and originating from six conflict countries. Focus group discussion participants (11 groups; 77 participants) included health, protection and community service staff working in the urban or camp settings. Interviews and discussions were conducted in the language of preference, with assistance by interpreters when needed, and transcribed for analysis by grounded-theory technique.

**Results:**

Single and multiple counts of GBV were reported and ranged from psychological and social violence; rape, gang rape, sexual coercion, and other sexual violence; abduction; and physical violence. Domestic violence was predominantly reported to occur when participants were living in the host country. Opportunistic violence, often manifested by rape, occurred during transit when women depended on others to reach their destination. Abduction within the host country, and often across borders, highlighted the constant state of vulnerability of refugees. Barriers to reporting included perceived and experienced stigma in health settings and in the wider community, lack of awareness of services, and inability to protect children while mothers sought services.

**Conclusions:**

Findings demonstrate that GBV persists across the span of the refugee experience, though there is a transition in the range of perpetrators and types of GBV that are experienced. Further, survivors experience significant individual and system barriers to disclosure and service utilization. The findings suggest that routine GBV screening by skilled service providers offers a strategy to confidentially identify and refer survivors to needed services within refugee settings, potentially enabling survivors to overcome existing barriers.

## Background

Refugee agencies, as well as global health and human rights experts, have begun to direct considerable attention to the prevention and response to gender based violence (GBV). GBV is defined in the United Nations Declaration on the Elimination of Violence Against Women, as any act “that results in, or is likely to result in, physical, sexual or psychological harm or suffering to women, including threats of such acts, coercion or arbitrary deprivations of liberty, whether occurring in public or in private life…and should encompass, but not be limited to, acts of physical, sexual, and psychological violence in the family, community, or perpetrated or condoned by the State, wherever it occurs.” These acts include: spousal battery; sexual abuse, including of female children; dowry-related violence; rape, including marital rape; female genital mutilation/cutting and other traditional practices harmful to women; non-spousal violence; sexual violence related to exploitation; sexual harassment and intimidation at work, in school and elsewhere; trafficking in women; and forced prostitution” [1]. Among refugees and internally displaced populations (IDPs), women and girls are particularly vulnerable to GBV such as forced sex/rape, early or forced marriage, abuse by an intimate partner, child sexual abuse, forced or coerced prostitution and sex trafficking [2,3]. A myriad of risk factors and situational contexts, ranging from the individual to structural level, increase the vulnerability of displaced persons. These include, but are not limited to: breakdown of social, family, and government protective structures; loss of or poor police protection, legal recourse, or justice; gender or ethnic discrimination; social acceptance of GBV; lack/loss of basic resources and economic disparity/loss; and low awareness of rights [4]. In response, UN agencies have formed an Inter-Agency Standing Committee to provide enhanced and coordinated efforts to end GBV among refugees and displaced populations and mitigate the potential long-term physical, mental and reproductive health and social issues that result [5].

Evidence of immediate and long-term physical, psychological, reproductive and social harms of GBV is extensive. Physical sequelae may include bodily and genital injury [3,6,7], pelvic pain, traumatic fistulas [8,9], unwanted pregnancy, increased risk of HIV [9,10] and sexually transmitted infections [11,12], and even death [13]. Adolescents and young women and those without prior sexual intercourse experiences are particularly vulnerable to physical trauma and genital-anal injury associated with sexual violence [6,9,14-16]. Physical injury may be worsened in cases in which there is a greater length of time to physical examination [16]. Likewise, post-exposure prophylaxis for HIV prevention may not be possible if examination and care is provided greater than 72 hours after sexual violence [17]. In contexts where STIs/HIV are prevalent among the population, sexual violence may increase transmission through unprotected oral, anal, and vaginal trauma by perpetrators as additional factors in STI/HIV transmission [10,18]. A recent global review of behavioral research demonstrated an increased association of HIV and HIV-related risk behaviors with coerced or forced sex in low- and middle-income countries [19]. Furthermore, mathematical modeling has demonstrated the reduction in incident infections in Kenya with reduced prevalence of sexual violence against high risk women [20]. Adverse mental health outcomes associated with GBV may include post-traumatic stress disorder, depression and suicidal thoughts, or substance abuse [13,21,22]. Social and familial stigma and rejection secondary to GBV may exacerbate mental health outcomes experienced by survivors [9].

Despite the enormity of the problem and the recognized need for services for survivors of GBV, understanding the burden of GBV among women in refugee and displaced populations remains elusive, particularly due to challenges in data collection methods [23]. Most estimates of GBV in complex humanitarian settings are based on self-reported experiences, which underscore the issue of underestimation of the true scope of the problem. Fear, stigma and discrimination further compound any reliable estimate of GBV [24]. The United Nations High Commissioner for Refugees (UNHCR) and implementing partner agencies have taken steps to enhance their capacity to respond to GBV, including providing comprehensive services for GBV that include health, protection and psychosocial services [5,9] as well as developing more sophisticated tools to collect information about reported cases of GBV through the Gender Based Violence Information Management System [25]. Given that the majority of GBV services and reporting mechanisms are passive systems that rely on survivor-initiated reporting and service-seeking, developing a proactive approach to identifying survivors for early intervention and care has the potential to improve health and social outcomes for survivors and their families as well as strengthen GBV monitoring systems through identification of cases in humanitarian settings.

While screening methods to identify intimate partner violence have been used among some refugee populations [19,26], to our knowledge a brief, validated screening tool for GBV does not exist for use in refugee settings. In an effort to fill this gap, this study aimed to bring the broad definition of GBV into a functional GBV screening tool to be adapted for use in camp and urban refugee settings. Such a tool, when implemented by a skilled service provider, could confidentially identify an individual who has experienced one or more types of GBV and link the individual to comprehensive services that are available in these settings. Building on the findings of our systematic review of GBV among conflict-affected populations, [27] qualitative research was conducted with female refugees from diverse countries who were survivors of GBV as well as with health, protection, and psychosocial providers. We aimed to identify the range of GBV experienced by female refugees, perpetrators of GBV, and locations where GBV occurs among conflict affected populations. The results of this qualitative research will inform the development of a brief GBV screening tool to confidentially and effectively identify female survivors for appropriate and timely intervention including referrals for health, psychosocial support, protection and other services.

## Methods

In January – February 2011, qualitative research techniques were conducted to: 1) describe the multiple types, locations and perpetrators of GBV as experienced by female refugees (15 years and older) living in urban and camp settings in Ethiopia, and 2) explore current barriers to survivors’ reporting and service seeking behaviors in urban communities and camp settings where health, protection and social services are provided to refugee populations.

### Study site

Focus groups and individual interviews were conducted in Addis Ababa and three refugee camps in the Jijiga district of Ethiopia. Addis Ababa is the urban setting that houses the country offices for UNHCR, the Ethiopian government’s Administration for Refugee and Returnee Affairs (ARRA), and several partner organizations. Over 1,000 refugees from diverse countries including Democratic Republic of Congo (DRC), Burundi, Rwanda, Sudan, Somalia and Eritrea live in Addis Ababa. Most urban refugees resided in a camp prior to arrival in Addis Ababa, and were moved as a result of protection challenges (experience/risk of violence or stigma) in the camp, severe medical complications that cannot be addressed in the camp, or in preparation to relocate to another country. Jijiga district is located in northeast Ethiopia, along the border of Somalia and Somaliland and three refugee camps have been established in this district: Kebribeyah (est. 1991), Aw Barre (est. 2007), and Sheder (est. 2008). The three camps accommodate approximately 11,600 to 16,300 refugees per camp, collectively accommodating over a total of 41,500 refugees, as of June 2012 [28]. The majority of the residents in the camps are refugees from Somalia.

### Study populations

Table 1 displays participant characteristics per site and qualitative method.

**Table 1:** Qualitative participants characteristics

**In-depth interviews with survivors**			
**Locations and context**	**No. of participants**	**Represented country/ies of origin**	**Age range**
**Urban setting: Addis Ababa**	17	Burundi, DRC, Eritrea, Somalia, Sudan	15 - 48 yrs.
**Camp 1**	7	Somalia	23 - 43 yrs.
**Camp 2**	7	Somalia	17 - 39 yrs.
**Camp 3**	6	Somalia	20 - 38 yrs.
***Total interview participants***	***37***		
**Focus Group Discussions**			
**Locations and context**	**No. of groups (participants)**	**Represented service type or organization**	
**Urban setting: Addis Ababa**	2 (10)	Health services; Protection	
**Camp 1**	2 (22)	Women and youth organizations; GBV services and social work	
**Camp 2**	2 (14)	Women and youth organizations; GBV services and social work	
**Camp 3**	2 (16)	Women and youth organizations; GBV services and social work	
**Combined participants across camps**	3 (15)	Health services; Protection; GBV services	
***Total***	***11 (77)***		

*Survivors*: Trained GBV service providers working in partner agencies invited female survivors to participate in the study. Eligibility criteria required that participants be female, aged 15 years or older, have refugee status (documentation not required), reside in the selected urban (Addis Ababa) or camp-based setting (Jijiga area), and currently receive or have received GBV services from UNHCR’s implementing partner agencies. Prior or current use of GBV services was an important eligibility criteria for inclusion of the survivors, as this ensured the survivors were willing to discuss GBV and also that they had an ongoing relationship with services if they needed post-interview support. To respect the sensitivity of the topic, individuals were not required to disclose any history of GBV at eligibility screening.

The target sample size for survivor interviews was a maximum of 20 participants in the urban setting and 10 per camp (total 50), estimated with an aim to obtain variation in the sample. Enrollment for interviews ended early upon reaching saturation, when no new information was obtained from interviews [29]. Thus, a total of 37 in-depth interviews were conducted among eligible female refugees. To ensure privacy for the participants, only private individual interviews (no focus group discussions) were conducted with survivors. Participants in the urban refugee program were selected to represent a range of countries of origin, including the DRC, Burundi, Sudan, Eritrea, and Somalia, while those from the Jijiga camps originated from Somalia. Verbal consent was used in lieu of signed consent to further ensure participant privacy and security. All participants provided informed consent prior to participation in interviews, which were conducted by trained researchers with support from skilled interpreters.

#### Service providers and community organizations

Eligibility criteria for focus group discussions with service providers and staff from community organizations required that participants be 18 years of age or older and worked at least one year for an agency/organization that provides services to refugee populations. Participants were purposively selected from the range of agencies and organizations in both the urban and camps settings and collectively included health providers, social workers, and protection officers. Participants were representatives of ARRA, the governmental body that provides assistance to refugees, several UN agencies, and international non-governmental organizations (INGO), national non-governmental organizations, and refugee organizations. Local women’s and youth groups were also included. A total of 11 focus groups (77 participants), ranging in size from 6–10 participants per group, were conducted with eligible service providers and community organization leaders. All participants provided verbal informed consent prior to participation in the focus groups. The consent process and group discussions were conducted by trained facilitators with support of translators, as needed.

### Measures and analysis

Semi-structured interview and discussion guides were developed in collaboration with partners and used when conducting the in-depth interviews and focus group discussions. The focus group discussions and interviews concentrated on several key areas for development and implementation of a GBV screening tool: 1) types and frequency of GBV experienced by refugees; 2) perpetrators of GBV; 3) location and context of GBV; and 4) barriers to reporting GBV and accessing services. Survivors and services providers were not asked to describe their personal experiences but to describe GBV among the refugee populations, in general; however, almost all survivors elected to share their personal experiences as examples to explain their responses.

Interviewers and focus group facilitators were trained to provide details on the study purpose, obtain verbal informed consent prior to data collection, and provide survivor participants with information about additional local GBV resources at the close of the interview. Focus groups and interviews ranged from 90 to 120 minutes in duration. Local, trained interpreters were hired for assistance to communicate with native Somali, French, Kiswahili, Tigrinya, and Amharic speakers. To further ensure confidentiality, camp-based interviews were interpreted by Somali-speaking staff hired from outside of the camps. No names or personal information was collected from any participant. Interviews and focus-group discussions were digitally recorded with permission and professionally transcribed. To check the quality of translation, several interviews were randomly selected for a second translation and transcription for comparison.

Data analysis followed a grounded theory approach, allowing for in-depth exploration of emerging themes in the participants’ narrative responses [30]. All English transcripts were entered into Atlas.ti software (Cincom Systems, Berlin) for coding. Each transcript was coded in duplicate by two research team members who met frequently to discuss themes and resolve any discrepancies in coding and themes. Topical codes were applied to allow quotations to be sorted according to interview guide domains and open interpretive coding was utilized to identify and analyze any emerging themes observed within and between topical areas.

Findings are presented by GBV type, locations and contexts, perpetrators, and barriers to reporting GBV. There was general agreement by service providers and survivors across themes, though some themes were discussed in more detail by providers as compared to survivors and vice versa; thus, findings from both groups are presented for each theme. Quotations were selected to highlight the themes developed from the analysis. A code that indicates participant type (‘GBV Survivor’ or ‘Service Provider FGD Participant’) and setting (‘Addis Ababa’ or ‘Camp’) follows each quotation so readers may identify the origin of the quotation. Some quotations are included to provide context to a particular theme and some may be relevant across multiple themes. Camp names and any other potential identifiers have been removed to protect the anonymity of study participants.

### Human subjects protection

The study was conducted in partnership with the UNHCR and ethical review and approval was provided by both ARRA, the governmental agency responsible for all refugee related concerns in Ethiopia, and the Johns Hopkins Medical Institutes Institutional Review Board (IRB No.: NA_00042672).

## Results

A range of violence types, perpetrators and contexts were described by participants and presented in further detail below. Table 2 displays the types of GBV and perpetrators identified in the locations where the experience of GBV occurred. The table does not represent prevalence of GBV but provides information on the GBV experiences of female refugees, contexts, and the groups who they identify as perpetrators. In many cases, survivors reported several types of GBV (e.g. physical, sexual, psychological) occurring in one incident with multiple perpetrators.

**Table 2:** Reported GBV experiences: Types of violence and perpetrators, by reported location of incident

	**Location where violence was reported to occur**		
**Violence construct**	**Country of origin**	** Transit to or within host country**	**Host country – urban and camp settings**
**Psychological violence**	**General threats of violence or rape by armed actors, threats against family**	** Coercion for assistance with transportation or shelter**	**Threats of violence, kidnapping**
	- Armed actors	- Strangers, acquaintances	- Intimate partner, family, family of partner (based in host country, other displaced setting, or country of origin)
	**Forced witness of murder rape, including that of family members or neighbors**	** Forced witness of rape of others in transit**	**Threats of withholding finances or food assistance**
		- Armed actors, strangers	- Intimate partner, employer
	- Armed actors		- NGO officer (camp)
**Type**	**Social stigmatization or isolation on the basis of single marital status, marital choice, FGM choice**		**Coercion for assistance with finances, food assistance, or shelter**
**-**Perpetrator(s)			
	- Home community, family, religious affiliates		- Intimate partner, employer
			- Other male refugee camp resident,
	**Social stigmatization or isolation on the basis of sexual violence experience**		**Social stigmatization or isolation on the basis of single marital status, marital choice, FGM choice**
	- Home community, family, religious affiliates		- Refugee community, family, religious affiliates
	**Social stigmatization or isolation with emphasis on religious, ethnic differences**		**Social stigmatization or isolation on the basis of sexual violence experience**
	- Armed actors, home community		- Community, family, religious affiliates, refugee community,
			**Social stigmatization or isolation due to religious, ethnic, clan differences**
			- Refugee community, host community
**Physical violence**	**Physical violence including beatings or torture**	** General physical violence,**	**General physical violence**
	- Armed actors, religious leaders or affiliates,	- Strangers,	- Strangers, family, partner's family
			-Host community, other refugee community member(s), other clan or ethnic group,
**Type**	**Imprisonment**		**Imprisonment**
**-**Perpetrator(s)	- Armed actors		- Strangers
	**Kidnapping/abduction**		**Kidnapping/abduction**
	-Armed actors, partner's family		- Strangers, partner's family
	**Intimate partner violence**		**Intimate partner violence**
	- Husband, ex-husband, boyfriend		- Husband, ex-husband, boyfriend
	**Other violence**		**Re-victimization after reporting or disclosure of GBV**
	- Family, partner's family, other ethnic group/clan		- Prior perpetrator (including but not limited to husband, ex-husband, boyfriend, neighbor/refugee community member)
	**Violence during pregnancy**		**Violence during pregnancy**
	- Armed actors		- Husband, ex-husband, boyfriend
**Sexual exploitation and violence**	**Coerced sex**	** Coerced sex**	**Coerced sex**
	- Armed actors	- Strangers (including those providing assistance with transit), acquaintances	- Strangers
			- Other refugee community member, employer, NGO staff member (camp)
**Type**	**Rape**	** Rape**	**Rape**
**-**Perpetrator(s)	-Armed actors, family or partner's family, stranger, religious leader or affiliate, other community member	- Armed actors, strangers (including those providing assistance with transit)	- Intimate partner, family or partner's family member, stranger, religious leader or affiliate, other neighbor or community member.
	**Gang rape**	** Gang rape**	-Host community member, religious leader or affiliate, employer, NGO staff leader (camp)
	- Armed actors	- Armed actors	
	**Rape during pregnancy**		
	- Armed actors		
	**Widow inheritance**		**Gang rape**
	- Husband’s family, home community		- Strangers
	**Unwanted sexual touching**-		**Unwanted sexual touching**
	Armed actors, family or partner's family, stranger, religious leader or affiliate, other community member		- Intimate partner, family or partner's family member, stranger, religious leader or affiliate, other neighbor or community member.
			**Financial control**
			- Husband, boyfriend
**Other violence types**	**FGM or pressure to complete FGM on child**		**FGM or pressure to complete FGM on child**
	- Home community, family, religious affiliates		- Refugee community, family, partner's family, religious affiliates
			**Early or forced marriage**
**-**Perpetrator(s)			- Pressure by family, extended family, refugee community (camp)

### Types of violence

Survivors reported multiple forms of GBV including physical violence, abductions, forced imprisonment, sexual violence, early or forced marriage, and social violence such as community-level stigmatization, threats, or isolation of a woman.

There are also many other different types of violence that women experience, like rape and sometimes forced sex from your husband. Sometimes also, early marriage, because we don’t have any choice. Our parents have to make the decision of our marriage. - Somali GBV survivor, Addis Ababa

Sexual violence was a common theme and included rape, multiple and gang rape, coerced sex, and other forms of sexual violence.

… when girls just go out of the camps, to fetch water, they threaten them to rape them right there, but we just tell them, okay, we just give you our ration. Please leave us, and then they just leave us alone, but unless we give them the ration, they will just run and then find us and then rape us. So threat is just used as one means. –Sudanese GBV survivor, Addis Ababa

Survivors also reported abduction, often combined with sexual and physical violence. One participant reported being abducted while pregnant and imprisoned by armed combatants for almost two years. She was abused and repeatedly raped while imprisoned in her home country of DRC. Following her escape, she and her infant son (who was born in captivity and also raped by combatants) eventually reached Ethiopia, where they were both diagnosed with HIV. Her second son, conceived in prison, was born in Ethiopia after her escape and was tested negative for HIV. Her experience in the ‘prison’ in DRC is recounted:

So one day I was at home, I was two months pregnant. Some people came in the house, my husband was at work. They came and they arrested me, they took me to the place, which I don't know. They started beating me; they said that me and my people, we are against them. I said, "Who are those people?" They are accusing me for being from Rwanda. I told them I'm not from Rwanda… They were beating me and I told them, "What you are doing? I'm pregnant." They are going to kill me and kill my child. And their [response] was like ‘that child is not __, he can die any time, we don't care about him. So they put me in that place, one of my neighbors called to my husband and told him that they raped me… At the place they put me, I don't know if it was prison or what, every night anyone can come and sleep with me. If I tell them that, "I'm pregnant, why are you doing this to me?" they will be beat me and do it by force. –Congolese GBV survivor, Addis Ababa

Service providers also reported similar types of GBV occurring in the camps or urban setting. Cases that were most often reported to health and protection officers did not necessarily reflect more prevalent forms of violence, but simply those that were most often reported by survivors or bystanders. Based in the urban area or the refugee camps, service providers’ experiences were often related to serving acute and recent cases that included: domestic violence; spousal control of finances, resources, or assistance; rape or abuse when leaving the camps to find firewood or other resources (see next section); and early or forced marriage. Domestic violence was often reported when neighbors overheard or witnessed the event.

[When asked what kinds of violence the service providers see in the camps:] *Domestic violence. Like hit them with a really big stick. And they beat them, or kick them… And the husband knows if she goes out and tries to find work, he's going to say to her, “Where were you? Were you being a slut? Like staying over there in the streets? What are you doing?” … There is also denial of resources. – Service Provider FGD Participant, Camp*

### Locations and contexts

GBV was reported to have occurred across multiple settings, including in the country of origin, during conflict or times of peace; the host country, within the urban or camp setting or within the host community; and during transit to or within the host country. Survivors reported experiences of GBV that occurred directly within the context of conflict while others reported that it was related to situations of increased vulnerability, secondary to conflict and displacement. Physical, sexual, and psychological violence types and perpetrators were similar across settings, except in two specific (though, not unexpected) situations: 1) armed actors were reported to be perpetrators in country of origin or during transit away from conflict, and 2) NGO and camp staff were identified to be among the perpetrators of GBV in the refugee camp settings. GBV that occurred during transit from country of origin to camps had less variation in types of violence and perpetrators than in the other contexts. The violations during transit were predominantly opportunistic, taking the forms of rape, coerced sex, and/or physical violence by strangers or by an acquaintance who took advantage of the displaced person’s increased vulnerability related to lack of shelter, transportation, or other basic needs.

…inside Congo now, before I decided to come. There were different people, sometimes you can meet soldiers and they took you in the camp, soldier camp, military camp and they can rape you there. I was raped by seven, seven people at the border. And once I was just -- I was unconscious, when I woke up I found myself full of blood. And from that day I have this bleeding problem. That way I had also this sexual transmitted disease. –Congolese GBV survivor, Addis Ababa

I remember a lot of women who were raped when I was in Somalia. Even when I was raped, I was not the only person who was raped on that truck. There were two other women who were raped with me. So it's not a new thing for me and it's not like I heard from the people. I actually faced it, I actually experienced it, and I saw other women who were raped in front of me and they get pregnant because of these things. I was raped in Somalia and then I run away from Somalia and I was raped while I was on my journey from Somalia to Ethiopia, and I entered the border and I was just newly [gave birth to] my child. – Somali GBV survivor, Addis Ababa

GBV occurred with slight variations across locations and settings, with respect to the types of perpetrators and level of accountability for committed acts of GBV. One participant highlighted the differences in violence between her home country and the host country:

[Here], if the woman or the girl has a boyfriend and has a relation, and she get pregnant without marriage, she will be excommunicated from her community, no one can talk to her, no one can shake her hands, no one can provide any help for her, like she is very dead to them. Here, it’s not like Somalia. In Somalia they might beat you and hurt you physically. But here, they can just want to stare you, and point to you, and assault you and talking behind you, but they cannot hurt you so much, because there is a government.– Somali GBV survivor, Addis Ababa

Close proximity and maintained familial and social networks in the country of origin also facilitated cross-border perpetrations of violence. This included, in particular, abductions and psychological forms of violence, including threats of abduction of the individual or the individual’s child(ren). Several participants in the camp and urban settings reported such experiences.

My daughter is sick, because when she was 16, some man saw her and he took her and she stayed a year with him. [Interviewer: How did he take her?] He said to her, “I love you and I want to marry you,” and he took her. But when he had sex, he just made her wash after… When she gave birth to one child, he kept the child and he kicked her out. [Interviewer: Was he from the camp?] Yes, at the time. He took her [the baby] to Somalia to give to his mother. – Somali GBV survivor, Camp

While individual characteristics, such as young age or being a single head of household, were significant vulnerabilities to GBV, physical surroundings played an important role in the ability of perpetrators to take advantage of a woman’s vulnerability. Many reports demonstrated the increased vulnerability of young girls when they were alone or, conversely, in densely populated areas such as weddings, where others may not immediately note their disappearance.

There is a lot of youth in the wedding. The perpetrator can just come in and nobody would notice. One of them is going to come up to the girl and say, "Can I talk to you?" and when he takes her into a place alone, they just kidnap her. Yeah, most of the cases are like this. – Somali GBV survivor, Camp 1

Likewise, vulnerabilities existed for single women who lived alone or with small children and/or where houses provided little structural protection, particularly in camp settings. We previously highlighted that women were coerced to have sex or become involved in prostitution; in some cases, sex was exploited in return for basic assistance for repairs to their houses. Other women acknowledged acquiescing to marriage simply for increased protection. The lack of other adult protection in the house or lack of physical protection offered by one’s housing structure may be observed by perpetrators, making these women a target for GBV, particularly in the camp setting.

On that evening when the wedding was done. There was someone who attacked [me in] my home [in the camp]… beating me up and like mercilessly and he is kicking me all over the place. I felt deeply unconscious this time it is like in coma and then in the morning I woke up and when the sun light like beaming sun rise I opened my eyes and see my daughter been also raped and also being tied up in the same way I was tied up that was like the biggest shock for me. – Somali GBV Survivor, Camp 2

For some participants, moving to a new location did not mean safety from violence; rather they were exposed to new risks for GBV. The following quotation details the experience of one survivor who reported being kidnapped and raped at the age of 14. She recounts the physical and psychological abuse she experienced after her release, when she moved to another town for safety, to avoid the stigma of rape, and to find employment.

While I was living here [in Addis Ababa] with my father and with my sister and then I was just having my life, just going to school and taking care of my sister, I was raped. I was kidnapped and then they raped me and then, later on, when my father and my brother, they came and it was like a fighting … and then my father tried to [tell] the boys that ‘she's under 18 and she is very young, she's 14 years old. She cannot be married to anyone and she has to live here with us’. So it was a fight… Then those men took them within that taxi. I was raped from all six men. I was living with them around about two months and my mother tried to follow me …later on, she found me. And my mother took me to her [town] because I can stay there and I can work as a housemaid from family to family so that I can get support myself. At the same time, I will not be hurt from the other family members because I was raped. And when my mom left me, I spent three years working from family to family but the problem is, the majority clan was living in there… they do have kind of like a revenge and they hate any person from my clan. So they were, hurting me, sometimes they would beat me. Sometimes they would cut my hair. I faced a lot of problems. My life is a mess. -Somali GBV survivor, Addis Ababa

As demonstrated in previous quotations, domestic violence was often linked to tension around financial assistance and rations provided to refugee families. The role of the men is often reversed when entering the refugee camp setting; they are no longer the breadwinners, rations are not given to men, and they cannot find employment due to governmental restrictions. This increases tensions in the home, may increase drug/alcohol use, and perpetuate domestic violence. Among some participants, this was often believed to be further complicated by the use of *khat*, a local plant that is chewed for its stimulant effects, for which family’s rations or financial assistance were sometimes used by the husbands, resulting in financial disparities and family dispute.

### Perpetrators

Perpetrators of physical, sexual, and psychological forms of violence included both unknown and known individuals. Individuals unknown to the survivor typically included armed actors and strangers in the host country or country of origin, or encountered in transit (described by prior quotations). In some cases, violence perpetrated by strangers was enabled by acquaintances.

I was raped. I was raped in Addis by this man, and they did send me for HIV and I returned positive and I have a child. I was raped because I was with my friends and then they just asked me to go with them to this man’s house. And then I thought that we were just going together, and then after I went and they all left, and then he forced me to have sex with him. He raped me…[I] was 15 years old. –GBV survivor, Addis Ababa

Perpetrators who were known to or trusted by the survivor included husbands, family members and relatives, neighbors and other camp residents, religious and authority figures, and NGO or humanitarian staff.

Sometimes women, when they are very sick and they don’t want to have sex, and they are very exhausted because of the children and taking care of the children, the husband will force them to have sex with them, and if she refuse, he will beat her and hurt her. He will beat her so much until she gets so weak, and then he will have sex with her forcefully. – Somali GBV survivor, Addis Ababa

With respect to perpetration by NGO or humanitarian staff, one participant detailed her experiences. This participant had survived significant, ongoing domestic violence and abuse by two previous husbands. She also reported coerced sex by an employer in an office affiliated with the camp. After reporting the incident the employee was transferred to another office.

The man I work for, with him, in the office, I’m cleaning the office [near the camp], and he forced me to have sex. “If you don’t allow me, you will not work.” So I said to him, I can’t leave this job…He said also to people who came to him [after it was reported], “She wants to start something in the office. That’s why. I didn’t ask her anything.”… But the head of this office, he said, “We’ll let him to go another office, so you can stay in the office to clean.” - Somali GBV survivor, Camp 3

### Barriers to reporting

For many reasons, GBV survivors decided not to report the violence to authorities.

Believe me, the [rape] numbers that [are known by] UNHCR, other organizations, and other refugee committees are small. It [the rape numbers] will be triple or even sometimes maybe double. There are people who are hiding it and not telling anybody. –Somali GBV survivor, Addis Ababa

GBV is a particularly sensitive issue and highly stigmatized among many cultures and hidden due to individual shame.

They would make excuse and say, “I was hit with a stone.” Or, “I fell.” Making excuses. Even if their neighbors know that she was beaten and she would deny it. Just one or two, or it's a like a lot of variety of things. [Facilitator: ‘Why do they deny it?’] She was ashamed to say, “My husband beat me.”–Service Provider FGD, Camp

Both survivors and service providers concurred that there was a concern among survivors that reporting could lead to others’ learning of their experience.

I went to the hospital in [camp] and I could not tell them what happened to me because there were some-- few families from Burundi were there, they know that my husband is in Addis. When I told this family or in the health center maybe they will tell my husband who is living in Addis that I was raped and I couldn't tell them, I kept secret. –Burundi GBV survivor, Addis Ababa

Many participants, survivors and providers alike, described the concern for physical violence and retribution by the perpetrator or others. Participants were concerned about lack of confidentiality and subsequent perceived or experienced negative reactions from family members and others in the community, whose reaction may be to (individually or collectively) shame, blame, or isolate the survivor. Survivors and providers agreed that social stigma prevented reports, particularly in case of sexual violence.

For example, she might not get the kind of trust from the person that she's come to tell. Maybe that person might tell to other people in order to insult. That's going to be one challenge. Another thing, you know, everybody will hurt her again saying that, “Oh, you've been raped. So you are kind of useless.”–Service Provider FGD, Camp

Some women feared repercussion in the host country if they reported the violence and named the perpetrators in their country of origin.

Most of the men who are raping the girls they are from Al-Shabaab group, and you cannot tell anybody that the one who did this to you is from Al-Shabaab, because they are religious people. You cannot say that Al-Shabaab raped me, because I remember one young Somali girl she was raped by two men from Al-Shabaab and when she came to the City and she told the people that she was raped by Al-Shabaab, immediately Al-Shabaab group in that City called her and they asked her family to dig a hole, and they put her there in that hole, and they asked everyone to throw a very heavy stone at her until she died. So even they will not kill you in a very good way, they do it so that you will feel not feel a lot of pain, like shooting you. They will kill you so will suffer and have a bleeding and then you will die.–Somali GBV survivor, Addis Ababa

Some women had concerns about dual stigma that included stigma against people living with (or presumed to be living with) HIV or AIDS as well as for having experienced GBV.

…they [the community] said to me like ‘you have AIDS, you were raped’ and then I told them ‘there is test let us go there to see. I was raped, that is true, but you are a woman like me. It is unluckily and it is an accident. I don’t have AIDS and you should not insult me like this.’ –Somali GBV survivor, Camp 2

Single women were particularly concerned with being blamed for sexual violence and labeled as promiscuous or being the ‘cause’ of a perpetrator’s infidelity. Unmarried and married women were also concerned with their future value as a wife and for the social well-being of their children, should others learn of their GBV experience.

I don't tell them even if they ask me. I get scared and I don't tell them that my daughter doesn't have a father. Because I am sure that they cannot provide any help to me regarding this issue so I prefer to keep quiet than tell them without doing anything. If I even get a trustful person, I feel, if I told them my story, that they will just be surprised or shocked and they cannot keep this secret from others, they will try to share it with other people. So I prefer to keep it to myself and don't tell anybody. – GBV Survivor, Addis Ababa

Traditional practices that require unmarried rape survivors to marry the perpetrator persist in some camps and were a barrier to reporting for some female survivors.

There is this incident two months ago a girl who was raped and after that she never reported anything like that and then she went home after she became pregnant and after that she told the mother there is rule that if you rape the girl you are going to marry her. They married the rapist to the girl…this happened in [camp]. – Somali GBV survivor, Camp 2

Women’s beliefs that husbands have the right to abuse their wives and their lack of awareness of their rights appeared to prevent women from reporting marital rape and intimate partner violence. Some survivors believed that GBV is a normal aspect of life for most women and thus does not warrant further attention; for others, shame and isolation led to the belief that the survivor was the only person who had experienced GBV, both acting as barriers to disclosing GBV and service seeking behaviors.

…[I think] am I the only woman, only girl who was raped and was pregnant and underage? And for me it was a shame. I couldn’t tell anybody this. That’s why I keep it as a secret myself.–Congolese GBV survivor, Addis Ababa

Lack of awareness of health risks related to untreated GBV, of rights to services, and of available services for GBV also created barriers to reporting. Some survivors believed that protection and justice against perpetrators were the only purposes of reporting an experience of GBV. Past experiences of substandard treatment by the justice system or lack of confidence in the system have prompted many women not to report their GBV experience. Many women feel reporting to be unwise given the potential stigma and the negative impact on themselves and their families from reporting such events.

Yeah and because I live in this camp, because of that, I had second thoughts. I wouldn’t have regretted it if the NGO had helped me to have my daughter’s card. If they did anything like that, I wouldn’t have regretted. But, since I am now being threatened to be out of the camp, it’s my problem. It’s going to be my problem. I’m going to have to face and deal with this problem. –Somali GBV survivor, Camp 2

Other women reported being treated only for the physical outcome of the violence, but receiving no further care or follow up. In medical settings, survivors were symptomatically treated and were not questioned about any experience of GBV or the reason for trauma. Clinic staff concurred but attributed the lack of GBV screening to time constraints, limited training and resources.

..*when I went in hospital, they only closed my head [wound]. Then after that, they give me some painkillers and they said to me, “You have to leave, have to go home.”…The people in the hospital, they only gave me medication, or some painkiller. They didn’t even check me if I have other problem- only my head.-Somali GBV survivor, Camp 3*

*Yes, I know for me personally, the number of patients I see in a day is great, so I just treat the emergency and give the medicine. You know, the clinician to patient ratio is just too low- that is one of the problems. And the other may be lack of awareness. Even with providers there is just lack of awareness. There is no kind of training [about] GBV*. *–Service Provider FGD, Camp*

Finally, basic issues of lack of transportation, money and language barriers were further barriers to reporting and seeking services. The vulnerability of children and risk that children could be kidnapped while women were away from the home also prevented women from seeking care as they were apprehensive to leave children alone or in the care of others.

Some women they can go to meet with [name of organization], but it’s not easy to talk with a protection officer; you will need to have an interpreter and sometimes we cannot have enough assistance with enough money to give that translator money so that he can interpret for the officers. At the same time, while we are going to complain or to tell about our problems to the officers or to health cares, we have another problem inside our homes, like my daughter or my son is six, so if I left him, who will take care of him? Someone can come after me and take my child from me, I might lose many things while I’m going there to complain. And when I would complain, I never ever be seen any reaction or any action was taken; helpful action which might help you to stop this violence, except separating the assistance between the wife and the husband, which is itself a problem. – GBV survivor, Addis Ababa

## Discussion

The purpose of this research was to understand the types, perpetrators, and contexts of GBV experienced by female refugees in multiple settings that should be included in a screening tool to confidentially identify survivors and barriers to effectively respond to GBV in humanitarian settings. Among urban and camp-based refugee populations, findings reveal multiple types of violence, settings and contexts in which violence occurs, and a range of perpetrators. Reports, provided by survivors and service providers alike, included a range of physical, sexual, and psychological violence and concur with other findings of GBV reported by female refugee and IDPs in other studies [2-4,8,31,32]. While other studies have provided evidence of abduction during conflict by armed combatants [33], also reported here, these findings are among the few to document the experience of abduction and threats of abduction within the host country or across borders from the host country humanitarian setting into the country of origin. These findings highlight the need for ongoing protection and attention to risks, even when refugees are considered to be settled in a secure setting.

Research efforts specific to GBV among refugees have commonly focused on either the experience of GBV in conflict [21,23,32-35] or mental health outcomes [35-38]. Here, we investigate GBV that occurs across the continuum of the refugee experience and whether GBV was reported to obtain justice and/or to access GBV services. Evidence is suggestive that even after escaping rape and physical violence perpetrated in conflict settings, women may continue to be subjected to GBV, such as domestic violence and sexual violence perpetrated by intimate partners, neighbors or other trusted individuals in the camp or urban host setting. Similar observations have quantified temporal transitions among East Timor refugees; for example in the post-conflict period, there was a 75% reduction in violence perpetrated by individuals outside of the family while some levels of IPV remained consistent or increased [39]. Our qualitative research suggests that the change in gender and family roles that occur with displacement, such as the husband’s loss of productivity and financial and community status, may escalate violence in an abusive relationship or contribute to the acceptance of domestic violence observed in refugee camp settings. Experiences of violence may vary according to the length of time a participant has resided in the camp or undergone displacement, and response efforts should be able to identify and adapt to these variations. As a result, services need to be comprehensive, for example able to respond to the multiple reproductive and mental health needs of women who experience gang rape as well as capable of providing protection and timely health services to women who disclose violence by their husbands in the camp setting.

Within humanitarian settings, GBV response is predominantly governed by several sets of guidelines, including but not limited to the *Inter Agency Standing Committee’s Guidelines on Guidelines for Gender-based Violence Interventions*[40]*.* For GBV, key steps include coordination and mapping of services and making reproductive health supplies available, [40] providing post-exposure prophylaxis (PEP) in areas with HIV prevalence greater than 1%, [41] and ensuring availability of counseling services. Training on GBV and human rights are included in guidelines for response. Further, humanitarian workers are trained and required to adhere to policies to prevent gender-based violence, including sexual exploitation and abuse. Monitoring and evaluation provides the feedback loop to assess trends and gaps in prevention and response [40]. During these times of increased attention on GBV due to consistently high levels of GBV in humanitarian settings, low access to services by those who experience GBV, [42] and faced by funding restrictions and fiscal challenges, implementers may find greater success in the use of evidenced-based interventions.

A review of studies on refugee camp density and size shows that camp design may help to mitigate structural vulnerability to GBV [43], such as reported here. Other proven GBV prevention strategies have been implemented in non-humanitarian settings. These include income enhancement and gender training, [44,45] male-targeted, community-based training sessions to address GBV and HIV risk behaviors, [46] training sessions with participatory learning and communications skills, [44,45] and community mobilization (trial underway) [47]. Though still few in number, research to inform evidence-based response efforts has been implemented in humanitarian settings, including studies of psychological support interventions for rape survivors, [48] community-led mobile clinic and psychological support services for male and female survivors of GBV, [37] and support of women and families through economic empowerment by village-led microfinance (trial underway) [49]. The dearth of data on evidence-based prevention and response for GBV in humanitarian settings sets a research agenda to develop a base with which to inform programming to provide the most effective and efficient responses in times of limited resources.

We found that significant barriers exist to GBV reporting and service utilization in the study population. Under-reporting of GBV experiences and low uptake of available services by survivors challenge GBV prevention and response programs implemented in humanitarian settings. To actively and confidentially overcome the barriers to disclosing GBV and referral, we used these findings to develop a screening tool that is multi-dimensional and captures broad domains and constructs of GBV, which we are now testing for validity and feasibility of use. Six questions covering psychological violence and threats of violence, physical violence, sexual violence, forced pregnancy, and forced marriage were included in the screening tool. Additional questions to identify locations and perpetrators are included and can be used to inform referrals for health, protection, and psychosocial services. For example, understanding the location or perpetrator of a sexual violence event can quickly inform service providers as to whether the survivor is still at risk and in need of additional protection. Such a screening tool may be integrated into existing GBV referral and surveillance mechanisms. Given the broad definition of GBV, [1] this proposed screening tool would not identify all forms of GBV but is designed to capture those widely reported and likely needing response from the health, psychosocial service providers, and/or protection officers. Other screening tools may be better positioned to identify other forms of GBV, for example, screening of female children may include questions to identify and respond to risk or recent experience of early marriage and female genital mutilation/cutting.

Like existing screening tools for intimate partner violence (IPV) or domestic violence, the proposed GBV screening tool for refugees may be implemented within the health settings [50], or may be implemented where confidential interviews may be conducted by trained service providers with refugees and where appropriate referrals are accessible to survivors. As with other screening tools, careful consideration should be given the setting where it will be implemented; for example, the proposed screening tool should only be used where skilled providers and GBV services are available, and where confidentiality and protection can be assured. This could include, for example, camp registration, during child–parent tracing interviews, and nutritional programs. Additional benefits to GBV screening include linkages to other data collection and documentation that is needed for program planning [51]. Finally, routine screening of women can serve a secondary purpose of changing the social norms that currently sustain GBV and increasing awareness of rights and services for those who have experienced GBV.

### Limitations

The findings should be viewed in light of several limitations.

Men and children are known to experience substantial forms of violence [35,52-54]. These populations may have different vulnerabilities, experiences, and interpretations of violence; thus, separate research studies for these groups are warranted. The research presented here focuses on female populations; however; the authors are currently collaborating to develop a screening tool for refugee and displaced men and boys.

Research was conducted among female refugees residing in urban and camp-based settings in Ethiopia. Refugees are often referred from camps to the urban setting to address serious health or protection needs. To overcome this potential bias, we used purposive sampling methods to recruit and interview participants with a range of GBV experiences. Because we used qualitative methods, selected GBV survivors for interviews, and did not include the general refugee population for interviews, we did not establish estimates of the prevalence of GBV among the refugee population studied. Refugee camps on the Somali border were selected for research activities and the findings derived from those settings may be limited to the Somali refugee experience. Sites were selected on the basis of several logistical, security, and ethical considerations (e.g. ensuring that established and quality referral services are available when survivors are identified). To ensure a range of experiences were captured, attention was given to include refugees from other countries (Sudan, Eritrea, Burundi, and DRC) in the interviews conducted in Addis Ababa.

An additional limitation is associated with the recruitment and eligibility of survivor participants. Survivors were recruited from the population of female refugees (15 years and older) who had reported their case of GBV and were receiving/had received GBV services from our implementing partners. Thus, these individuals had successfully overcome many of the barriers described above. Experiences and barriers reported in this manuscript may, therefore, be different among those who were not receiving services or had not disclosed GBV to service providers. This recruitment method and eligibility criteria, however, was identified in collaboration with the implementing partners working in the camps as ethically appropriate as talking about the GBV can be distressing, therefore, the survivors invited and consented to participate were prepared to discuss GBV and had ongoing access to services.

## Conclusions

The study demonstrates the existence, similarities, and variations of multiple types of GBV and perpetrators across the span of the female refugee experience in Ethiopia. These experiences range from, but are not limited to, physical violence and rape during conflict, opportunistic violence during transit, and sexual and domestic violence within urban and camp-based settings. Findings suggest a need for flexibility of programs to identify, address, respond, and prevent the range of GBV, which female refugees may experience. Additional findings highlight under-reporting and low service utilization by survivors. Informed by qualitative descriptions, we have developed a screening tool to confidentially identify and refer GBV survivors to appropriate and timely services within the humanitarian setting. Validation and implementation studies will provide an evidence-base for the use of the screening tool in humanitarian settings.

## Abbreviations

ARRA: Administration for Refugee and Returnee Affairs; DRC: Democratic Republic of Congo; GBV: Gender-based violence; IPV: Intimate Partner Violence; INGO: International Non-governmental Organization; NGO: Non-governmental Organization; UNHCR: United Nations High Commissioner for Refugees.

## Competing interests

The authors declare that they have no competing interests.

## Authors’ contributions

AV, LR, NG, AW, KP conceived of the study and designed the protocol. NG, AW and AA led in-depth interviews; AV, KP, LR, led focus group discussions; AW and two Masters-level student coders conducted qualitative coding (RG, LB); AW provided analysis, interpretation of the results, and drafting of the paper. AA participated in the management of field level data collection. All authors have read, provided input, and approved the final manuscript.

## Authors’ information

Qualitative research presented here is part of a multi-phase study conducted by the research team (NG, KP, LR, AW, AV) to develop and validate an evidence-based screening tool to identify and refer female and male survivors of sexual and gender-based violence in humanitarian settings. The tool is being developed among refugees and displaced populations in Ethiopia, Colombia, and Uganda with the ultimate aim for international use by UNHCR and other providers to confidentially identify and meet clinical, reproductive health, and mental health care needs and provide protection for survivors.
